# A Correlation-Based Joint CFAR Detector Using Adaptively-Truncated Statistics in SAR Imagery

**DOI:** 10.3390/s17040686

**Published:** 2017-03-27

**Authors:** Jiaqiu Ai, Xuezhi Yang, Fang Zhou, Zhangyu Dong, Lu Jia, He Yan

**Affiliations:** 1 School of Computer and Information, Hefei University of Technology, Tunxi Road, Hefei 230009, China; xzyang@hfut.edu.cn (X.Y.); zhoufang@hfut.edu.cn (F.Z.); dzyhfut@hfut.edu.cn (Z.D.); LuJia@hfut.edu.cn (L.J.); 2 College of Electronic and Information Engineering, Nanjing University of Aeronautics and Astronautics, Yudao Avenue, Nanjing 210016, China; yanhe@nuaa.edu.cn

**Keywords:** SAR, ship detection, correlation-based joint CFAR, 2D joint log-normal distribution, adaptively truncated clutter statistics

## Abstract

Traditional constant false alarm rate (CFAR) detectors only use the contrast information between ship targets and clutter, and they suffer probability of detection (PD) degradation in multiple target situations. This paper proposes a correlation-based joint CFAR detector using adaptively-truncated statistics (hereafter called TS-2DLNCFAR) in SAR images. The proposed joint CFAR detector exploits the gray intensity correlation characteristics by building a two-dimensional (2D) joint log-normal model as the joint distribution (JPDF) of the clutter, so joint CFAR detection is realized. Inspired by the CFAR detection methodology, we design an adaptive threshold-based clutter truncation method to eliminate the high-intensity outliers, such as interfering ship targets, side-lobes, and ghosts in the background window, whereas the real clutter samples are preserved to the largest degree. A 2D joint log-normal model is accurately built using the adaptively-truncated clutter through simple parameter estimation, so the joint CFAR detection performance is greatly improved. Compared with traditional CFAR detectors, the proposed TS-2DLNCFAR detector achieves a high PD and a low false alarm rate (FAR) in multiple target situations. The superiority of the proposed TS-2DLNCFAR detector is validated on the multi-look Envisat-ASAR and TerraSAR-X data.

## 1. Introduction

Synthetic aperture radar (SAR) is an active radar that can provide high-resolution images in the microwave band under all weather conditions. SAR images are less influenced by the time and weather conditions than the optical images, therefore, they are more suitable for ship detection. Nowadays, SAR images have been widely used for fishing vessel detection, ship traffic monitoring and immigration control [1,2,3,4,5,6].

The well-known constant false alarm rate (CFAR) detectors adaptively calculate the detection threshold based on parameter estimation and precise modeling of the background clutter. The statistical model of the clutter is usually accomplished by local parameter estimation of a moving reference window which is divided into the test window, the guard window, and the background window. For the simplest case, when the test window only comprises one pixel, we only have to estimate the statistical distribution of the clutter. The statistical models have been extensively studied in the past decades, and quite a number of models, such as log-normal [7], Weibull [8], generalized Gamma [9], K [10], alpha-stable [11], and G model [12], etc., have been developed. CFAR detection is realized to compare the pixels in the test window with the local calculated threshold. The CFAR detectors based on K (K-CFAR), G, alpha-stable, and generalized Gamma, etc., suffer complex parameter estimation and heavy computational burden. However, cell-averaging CFAR (CA-CFAR) [13] and two-parameter CFAR (abbreviated as NM-CFAR in the intensity domain, and LN-CFAR in the log-intensity domain) [14] are widely used for simple parameter estimation and high computational efficiency. CA-CFAR uses negative exponential distribution for single-look images and gamma distribution for multi-look images. CA-CFAR only estimates the mean value, however, while NM-CFAR and LN-CFAR assume that the statistical distribution of the clutter is Gaussian, and they estimate two parameters (mean and standard deviation) in the intensity domain and the log-intensity domain, respectively. CA-CFAR has good performance in homogeneous state, and two parameter CFAR performs well in non-homogeneous state. When the test window encompasses multiple pixels (e.g., nine pixels for a 3 × 3 test window), we need to estimate the JPDF of the clutter, which is a much more complicated task. However, in SAR images, every pixel of the clutter is correlated with its neighborhood, and the same to the target. Thus, in the simplest way, we can exploit the correlation information between neighboring pixels and model their JPDF. The first author of this paper, Ai [15], built a 2D joint log-normal (2DLN) distribution to model the JPDF of the clutter and, thus, correlation-based joint CFAR detection (2DLN-CFAR) is realized.

As for statistical modeling, the statistical models are established using the statistics estimated from the local background window. However, in practice, the clutter in the local background window is often contaminated by the high-intensity outliers such as interfering ship targets, side-lobes, and ghosts caused by the low pulse repetition frequency (PRF). The high-intensity outliers in the background window lead to parameter overestimation, which causes inaccurate statistical modeling. Consequently, the FAR drops, but the PD degrades, known as the *capture effect*. Numerous studies [16,17,18,19,20,21,22,23,24,25,26,27,28,29,30,31,32,33,34,35,36,37,38,39,40,41,42,43,44] have been carried out by means of extracting the clutter pixels in the background window and eliminating the influence of the high-intensity outliers. The order statistic CFAR (OS-CFAR) [16] is designed to overcome the above problem arisen in CA-CFAR. OS-CFAR has a significant advantage when detecting targets in multiple target situations, but the optimal statistic is obtained by experience and the computational efficiency is quite low. The smallest-of CFAR (SO-CFAR) [17] and the greatest-of CFAR (GO-CFAR) [18] choose the smallest and the largest mean value of the divided background windows as the local detection threshold. They both achieve improved detection performance in the background containing high-intensity outliers, but GO-CFAR suffers PD degradation and SO-CFAR suffers an increased FAR. The variability index CFAR (VI-CFAR) [19] dynamically selects a particular group of reference pixels for parameter estimation, and the test statistic is selected by a comprehensive method using CA-CFAR, SO-CFAR, and GO-CFAR. It performs well both in homogeneous and non-homogeneous backgrounds, but it suffers a detection loss in heterogeneous clutter. The trimmed mean CFAR [20] obtains the detection threshold using the mean value of the intensity ranked samples, but the optimum detection performance relies on the selection of the trimmed samples. The region classification CFAR (RC-CFAR) [21] subdivides the reference cell into four parts so that the number of target samples in each part becomes too small, thus, it is less credible to judge whether the background is non-homogeneous. The automatic censoring scheme [22,23,24,25,26,27,28,29,30,31,32,33,34,35,36] excludes the interfering outliers from the background clutter, so the statistical model is more accurate and the capture effect can be greatly alleviated. However, it is realized through additional iterative data censoring, which generally requires many cycles and long calculation time. Moreover, a method based on sub-aperture cross-correlation magnitude is proposed in [37], then the sub-band extraction strategy is further studied in [38]. However, sub-aperture processing in such methods degrades the spatial resolution and is unfavorable to detect small ships. A modified CFAR algorithm based on object proposals [39] uses an object proposal generator based on gradient features to generate a small set of object proposals. However, it spends much time on the object proposal generation, and the CFAR detection accuracy is affected by the object proposal procedure. Superpixel-based CFAR [40] incorporates the superpixel approach into the two-parameter CFAR detector. Superpixel approach-based segmentation is firstly implemented, and then two-parameter CFAR is applied on the segmented regions. Although it achieves a good result in multiple target situations, it suffers a heavy computational burden on the superpixel approach-based segmentation. In addition, there are many hybrid CFAR detectors designed to accommodate reversal clutter backgrounds in one algorithm. These CFAR detectors incorporate different strategies and dynamically choose the appropriate one. One classical example is the censored mean-level detector (CMLD) [41], which employs both data ranking and censoring methods to obtain improved performance in the presence of interfering targets. It excludes the largest reference sample and uses the remaining for parameter estimation. CMLD is quite robust in multiple-target situations, but it suffers some detection loss in homogeneous state. Moreover, without prior knowledge of the interfering targets, it may lose its robustness. The truncated statistics-based CFAR detector (TS-CFAR) [42,43] ranks the pixels in the background window based on gray intensity in a descending order. Then a truncation depth is set empirically (such as 25% in the experiments), and the high-intensity samples are discarded. Finally, the detection threshold is calculated using the remaining low-intensity samples. Furthermore, the same author of [43] proposed a segmentation-based CFAR detection algorithm using truncated statistics [44], segmentation is firstly implemented on the SAR images, and then truncated statistics-based CFAR is applied to the segmented regions. However, parameter estimation and threshold calculation in [42,43,44] need iterative numerical solutions, which are computationally ineffective. Moreover, the truncated statistics based CFAR detectors improve the PD, but the FAR rises because of the exclusion of the high-intensity clutter pixels from the background samples.

Traditional CFAR detectors simply use the contrast information between ship targets and the clutter, but the gray intensity correlation information is ignored. Furthermore, in multiple target situations, improved CFAR detectors are designed to enhance the PD through clutter extraction. However, the clutter samples are acquired through iterative data censoring or clutter trimming, the former needs many cycles and long calculation time, and the latter suffers a high FAR because the clutter samples are incomplete. This paper proposes a correlation-based joint CFAR detector using adaptively-truncated clutter statistics in SAR imagery. Compared with traditional CFAR detectors, our main contribution is stated as follows:

(1) In addition to the contrast information between ship targets and the clutter, the proposed joint CFAR detector exploits the gray intensity correlation characteristics in the clutter and ship targets. Joint CFAR detection is realized by building a 2D joint log-normal model as the JPDF of the clutter. 2DLN-CFAR [15] only exploits the gray intensity correlation of the eight-neighborhood, this paper extends it to a wider neighborhood, and more correlation information can be used to further lower the FAR, while the PD is obtained at a high level.
(2) In multiple target situations, 2DLN-CFAR and traditional CFAR detectors suffer PD degradation. Inspired by the CFAR detection methodology, the proposed CFAR detector designs an adaptive threshold-based clutter truncation method to eliminate the high-intensity outliers from the clutter samples in the background window, and the real clutter is preserved to the largest degree. The threshold is adaptively calculated by comprehensive consideration of real clutter preservation and high-intensity outlier elimination.
(3) A 2D joint log-normal model is accurately built using the adaptively-truncated clutter by simple parameter estimation, so the PD of the joint CFAR detector is greatly improved. The proposed TS-2DLNCFAR detector achieves a high PD and a low FAR, which can greatly eliminate the capture effect in multiple target situations.

The rest of the paper is organized as follows: In Section 2, the correlation-based joint CFAR detection methodology is introduced. In Section 3, the proposed TS-2DLNCFAR detector is detailed. In Section 4, experimental results are given with detailed analysis. Finally, Section 5 concludes this paper.

## 2. Correlation-Based Joint CFAR Detection

In SAR images, the gray intensity of ship targets is larger than that of the clutter because of the strong backscattering cross-section of the dihedral, trihedral, and polyhedral corner reflectors. Furthermore, every pixel’s gray intensity is correlated with its neighborhood. The first author of this paper, Ai [15], comprehensively used the contrast and the correlation information, and 2DLN distribution was built to model the JPDF of the clutter to realize joint CFAR detection. 2DLN model of the JPDF is defined as [15]:

(1) $$f(X,Y,\theta )=\frac{1}{2\pi \sigma_{ln}{}^{2}\sqrt{1-r_{\theta }{}^{2}}XY}\exp\left(-\frac{{\left(ln\left(X\right)-\mu_{ln}\right)}^{2}-2r_{\theta }\left(ln\left(X\right)-\mu_{ln}\right)\left(ln\left(Y\right)-\mu_{ln}\right)+{\left(ln\left(Y\right)-\mu_{ln}\right)}^{2}}{2\sqrt{1-r_{\theta }{}^{2}}\sigma_{ln}{}^{2}}\right)$$

where *μ_ln_* and *σ_ln_* are the mean and standard deviation of the local background window in the log-intensity domain. *X* and Y are the gray intensity of a pixel and its neighborhood in the direction of *θ* [15] uses a test window with a size of 3 × 3 pixels, and the gray intensity correlation in the four directions (horizontal, vertical, diagonal and anti-diagonal) are comprehensively used. *r_θ_* is the spatial correlation coefficient used for correlation evaluation, which is defined as [45]:

(2) $$r_{\theta }(l,k)=\frac{\sum_{i=1}^{M}\sum_{j=1}^{N}\left[f(i,j)-\mu ][f(i+l,j+k)-\mu \right]}{\sum_{i=1}^{M}\sum_{j=1}^{N}{\left[f(i,j)-\mu \right]}^{2}},$$

where μ is the mean value of the SAR image in the intensity domain, f(i,j) is the intensity value of the pixel located at (*i*, *j*), f(i+l,j+k)is the intensity value of its neighboring pixel located at (i+l,j+k). l and k are the distance values in the horizontal and vertical direction. (1) horizontal: *l* = 0, *k* = 1; (2) vertical: *l* = 1, *k* = 0; (3) diagonal: *l* = −1, *k* = 1; and (4) anti-diagonal: *l* = 1, *k* = 1. 2D joint CFAR detection can be realized using the joint CFAR detection threshold *T* calculated from the probability of false alarm (PFA) *P_FA_* by:

(3) $$P_{FA}=\int_{T}^{\infty }dy\int_{T}^{\infty }f(X,Y,\theta )dx$$

2D joint CFAR detection results in the four directions are acquired as follows:

(4) $$\begin{array}{l} \mathrm{Horizontal}:\;\mathrm{if}\;f(i,j)>T_{H}\;\mathrm{and}\;f(i,j+1)>T_{H},\;f_{dH}(i,j)=1\;\mathrm{and}\;f_{dH}(i,j+1)=1,\\ \mathrm{Vertical}:\mathrm{if}\;f(i,j)>T_{V}\;\mathrm{and}\;f(i+1,j)>T_{V},\;f_{dV}(i,j)=1\;\mathrm{and}\;f_{dV}(i+1,j)=1,\\ \mathrm{Diagonal}:\;\mathrm{if}\;f(i,j)>T_{D}\;\mathrm{and}\;f(i-1,j+1)>T_{D},\;f_{dD}(i,j)=1\;\mathrm{and}\;f_{dD}(i-1,j+1)=1,\\ \mathrm{Anti}-\mathrm{diagonal}:\;\mathrm{if}\;f(i,j)>T_{A}\;\mathrm{and}\;f(i+1,j+1)>T_{A},\;f_{dA}(i,j)=1\;\mathrm{and}\;f_{dA}(i+1,j+1)=1, \end{array}$$

where $T_{H}$, $T_{V}$, *T_D_* and $T_{A}$ are the joint CFAR detection thresholds in the horizontal, vertical, diagonal, and anti-diagonal direction, and $f_{dH}$, $f_{dV}$, $f_{dD}$ and $f_{dA}$ are the joint CFAR detection results in the horizontal, vertical, diagonal, and anti-diagonal directions. The points labeled as “1” are regarded as ship targets. The final joint CFAR detection result is obtained by fusing the four detection results with the “OR” operation [15].

**Figure 1:**
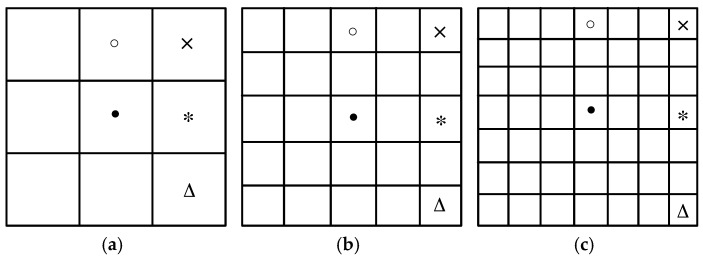
Different neighboring distances used for joint CFAR detection with a 7 × 7 test window: (**a**) one pixel; (**b**) two pixels; and (**c**) three pixels.

2DLN-CFAR [15] only uses the correlation information of the eight-neighborhood. However, in SAR images, ship targets are presented as a cluster of bright pixels with a certain length and width. In addition to the eight-neighborhood, we can exploit more correlation information in a wider neighborhood. Suppose the test window is 7 × 7 pixels, we can obtain the joint CFAR detection results with neighboring distances of 1, 2, and 3 pixels, as shown in Figure 1. We only use the correlation information in the horizontal, vertical, diagonal, and anti-diagonal directions instead of all directions to reduce the redundancy and improve the computational efficiency. The gray intensity of neighboring pixels with a certain distance are compared with the joint CFAR detection threshold calculated through Equation (3), and the joint CFAR detection results are acquired by Equation (4).

**Figure 2:**
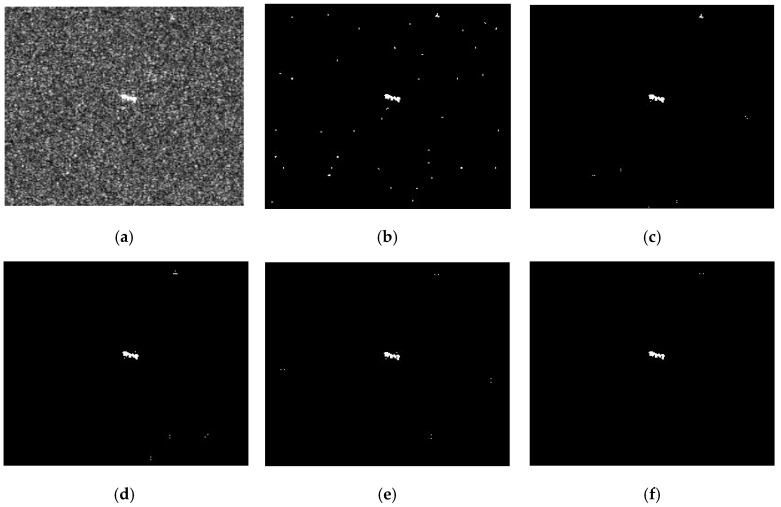
Joint CFAR detection results with different neighboring distances: (**a**) the TerraSAR-X image. One large ship and strong speckle are presented, (**b**–**e**) are the joint CFAR detection results with a neighboring distance of 1, 2, 3, and 4 pixels, respectively. They are obtained by fusing the results in the four directions using the “OR” operation. (**f**) is the joint CFAR detection result by fusing (**b**–**e**) using the “AND” operation. A 10^−4^ specified PFA is applied.

The joint CFAR detection result of a certain direction misses some information of the ship targets, so the “OR” operation is used to fuse the detection results in the four directions to improve the PD. However, the FAR is still high if we only use the correlation information of the eight-neighborhood, which is shown in Figure 2b. In SAR images, ship targets are presented as a cluster of bright pixels. However, the strong speckle is presented as isolated bright pixels. Moreover, they are randomly distributed. The above characteristics motivate us to exploit more correlation information of the ship targets in a wider neighborhood. A simulation is done to show this. Figure 2a is the TerraSAR-X image, one ship target and strong speckle are presented. Figure 2b–e is the joint CFAR detection results obtained with a neighboring distance of 1, 2, 3, and 4 pixels, respectively. The joint CFAR detection results are obtained by fusing the detection results in the four directions using the “OR” operation. Under different neighboring distances, the ship target can be detected, while the false alarms are randomly distributed. We can further fuse the detection results using the “AND” operation to lower the FAR, which is shown in Figure 2f.

Based on our previous work [15], the proposed TS-2DLNCFAR detector extends the eight-neighborhood to a wider one. Comprehensively considering a high PD and a low FAR, an optimal size of the test window can be selected, and the joint CFAR detection results with different neighboring distances are obtained using the “OR” operation. Finally, the “AND” operation is used to fuse the detection results with different neighboring distances. Undoubtedly, the proposed joint CFAR detection method can acquire a high PD and a low FAR.

## 3. The Proposed TS-2DLNCFAR Detector

**Figure 3:**
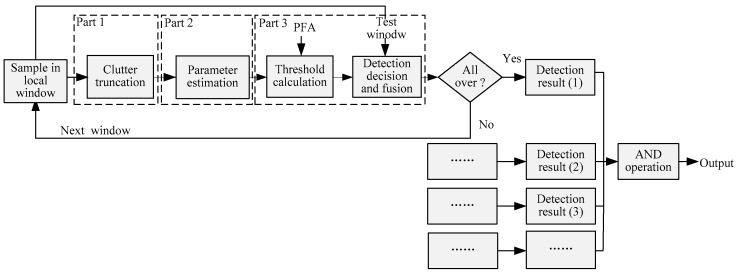
Joint CFAR detection flowchart of the proposed TS-2DLNCFAR detector.

Figure 3 illustrates the detection flowchart of the proposed TS-2DLNCFAR detector, which includes clutter truncation, parameter estimation, threshold calculation, joint CFAR detection decision, and fusion. TS-2DLNCFAR firstly applies clutter truncation in the background window. Joint CFAR detection results with different neighboring distances (one pixel neighboring distance is abbreviated as (1) in the flowchart, etc.) are acquired using the adaptively-truncated clutter statistics, and the final result is obtained by fusing the detection results with different neighboring distances using the “AND” operation. Each part will be introduced in detail in the later sections.

### 3.1. Adaptive Clutter Truncation in the Background Window

The proposed TS-2DLNCFAR uses log-normal as the statistical model of the clutter, which is defined as:

(5) $$p_{c}\left(x\right)=\frac{1}{\sqrt{2\pi }\sigma_{ln}\cdot x}\exp\left(-{\frac{\left(ln\left(x\right)-\mu_{ln}\right)}{2\sigma_{ln}{}^{2}}}^{2}\right)\;x>0$$

where *x* is the gray intensity of the clutter, $\mu_{ln}$ and $\sigma_{ln}$ is the mean and standard deviation of log-intensity ln(x). Their relation to the mean μ and standard deviation *σ* of intensity *x* is derived as:

(6) $$\mu =e^{\left(\mu_{ln}+\sigma_{ln}{}^{2}/2\right)},\;\sigma =\sqrt{e^{\left(2\mu_{ln}+\sigma_{ln}{}^{2}\right)}\cdot \left(e^{\sigma_{ln}{}^{2}}-1\right)}$$

Undoubtedly, the log-intensity value ln(x) follows the Gaussian distribution:

(7) $$p_{c}\left(y=ln\left(x\right)\right)=\frac{1}{\sqrt{2\pi }\sigma_{ln}}\exp\left(-{\frac{\left(y-\mu_{ln}\right)}{2\sigma_{ln}{}^{2}}}^{2}\right)$$

So, traditional CFAR detection based on a log-normal distribution can be regarded as:

(8) $$PFA=\int_{T}^{\infty }\frac{1}{\sqrt{2\pi }\sigma_{ln}\cdot x}\exp\left(-{\frac{\left(ln\left(x\right)-\mu_{ln}\right)}{2\sigma_{ln}{}^{2}}}^{2}\right)dx=\int_{ln\left(T\right)}^{\infty }\frac{1}{\sqrt{2\pi }\sigma_{ln}}\exp\left(-{\frac{\left(y-\mu_{ln}\right)}{2\sigma_{ln}{}^{2}}}^{2}\right)dy$$

where *PFA* is the probability of false alarm, so $z=\frac{ln\left(x\right)-\mu_{ln}}{\sigma_{ln}}$ obeys the standard normal distribution, and Equation (8) can be further expressed as:

(9) $$PFA=\int_{t}^{\infty }\frac{1}{\sqrt{2\pi }}\exp\left(-{\frac{z}{2}}^{2}\right)dz=\frac{1}{2}\int_{t/\sqrt{2}}^{\infty }\frac{2}{\sqrt{\pi }}\exp\left(-w^{2}\right)dw=\frac{1}{2}-\frac{1}{2}erf\left(\frac{t}{\sqrt{2}}\right)$$

where *t* is the scale factor, and erf(⋅) is the error function, which is defined as:

(10) $$erf\left(t\right)=\frac{2}{\sqrt{\pi }}\int_{0}^{t}\exp\left(-x^{2}\right)dx=1-\frac{2}{\sqrt{\pi }}\int_{t}^{\infty }\exp\left(-x^{2}\right)dx$$

Accordingly, for the pixel under test (PUT) with intensity $I_{0}$ in the test window, it is detected according to the following decision rule:

(11) $$z=\frac{ln\left(I_{0}\right)-\mu_{ln}}{\sigma_{ln}}\begin{array}{c} \overset{H_{1}}{>}\\ \underset{H_{0}}{\leq } \end{array}t$$

(12) $$ln\left(I_{0}\right)\begin{array}{c} \overset{H_{1}}{>}\\ \underset{H_{0}}{\leq } \end{array}\mu_{ln}+t\cdot \sigma_{ln}$$

where $H_{1}$ is the hypothesis that the PUT is a target pixel, and *H*_0_ is the hypothesis that the PUT is a clutter pixel.

The guard window in traditional CFAR detectors is set to avoid the interfering target contamination to the clutter samples in the background window. However, in practice, this is ineffective, especially in multiple target situations. The reference window of the proposed TS-2DLNCFAR only comprises a test window and a background window. Inspired by the CFAR detection methodology, the clutter samples in the background window are truncated using an adaptive threshold to eliminate the high-intensity outliers. The threshold is adaptive to the changing background clutter, as shown in Equation (12). The adaptive threshold based clutter truncation is detailed as follows:

- Calculate the mean $\mu_{B-ln}$ and standard deviation $\sigma_{B-ln}$ in the log-intensity domain using all samples of the background window.
- Truncate the clutter samples in the background window using an adaptive threshold. Suppose the gray intensity of a pixel in the background window is $I_{B}$, if it satisfies Equation (13), it will be excluded from the truncated clutter samples.
  (13) $$ln(I_{B})\geq \mu_{B-ln}+t_{1}\cdot \sigma_{B-ln}$$
  where $t_{1}$ is the truncation degree, which is vital to the performance of TS-2DLNCFAR. The lower $t_{1}$ is, the better the high-intensity outliers can be removed from the truncated clutter, but the more the real clutter is eliminated. Since the log-intensity of the clutter obeys the Gaussian distribution, using the adaptive threshold-based clutter truncation, the proportion of the real clutter preserved in all real clutter *Rc*1 is derived as:
  (14) $$Tc1=1-\int_{\mu_{ln}+t_{1}\cdot \sigma_{ln}}^{\infty }\frac{1}{\sqrt{2\pi }\sigma_{ln}}\exp\left(-{\frac{\left(x-\mu_{ln}\right)}{2\sigma_{ln}{}^{2}}}^{2}\right)dx=\frac{1}{2}+\frac{1}{2}erf\left(\frac{t_{1}}{\sqrt{2}}\right)$$

**Figure 4:**
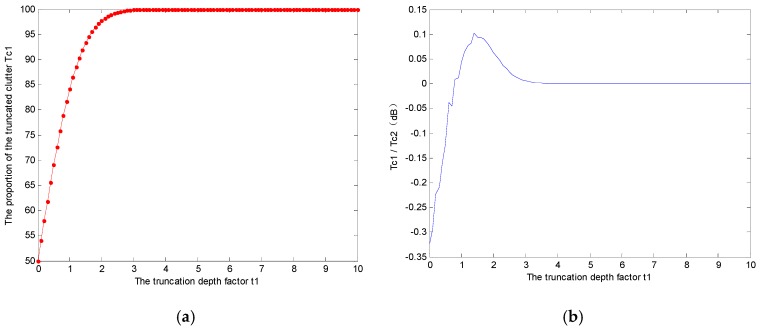
Real clutter preserving property analysis: (**a**) proportion of the preserved real clutter in all real clutter *Tc*1; and (**b**) *Tc*1/*Tc*2 value under different truncation degrees (in decibels).

A plot of *Tc*1 against $t_{1}$ is shown in Figure 4a. When $t_{1}\geq 1.3$, *Rc*1 is as high as 90%; when $t_{1}\geq 1.9$, 97% of the real clutter will be preserved; when $t_{1}\geq 2.3$, *Tc*1 approaches 99%. A test is implemented to show the good real clutter preserving capability of the adaptive threshold-based clutter truncation. The test selects a pure clutter region from the Envisat-ASAR image, and a plot of *Tc*1/*Tc*2 against $t_{1}$ is shown in Figure 4b. The real clutter preserving rate *Tc*2 is defined as the proportion of the preserved pixels in all pixels. From Figure 4b, we can reach the conclusion: *Tc*2 coincides with *Tc*1, the deviation is no more than 0.3 dB.

**Figure 5:**
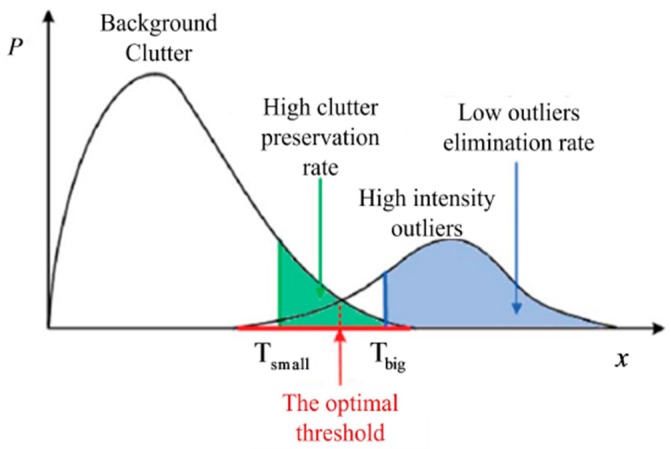
Optimal threshold selection.

Comprehensively considering real clutter preservation and high-intensity outlier elimination, the optimal value of $t_{1}$ should be selected as the intersection point of the distributions of the background clutter and the high-intensity outliers, which is illustrated in Figure 5. We can easily obtain the distribution of the background clutter, but the statistical distribution of the high-intensity outliers is difficult to obtain. Unfortunately, the optimal threshold is difficult to obtain using the intersection point. Nevertheless, inspired by the CFAR detection methodology, we can choose a relatively high real clutter preservation rate such as 97%; then $t_{1}$ can be adaptively calculated using Equation (14).

**Figure 6:**
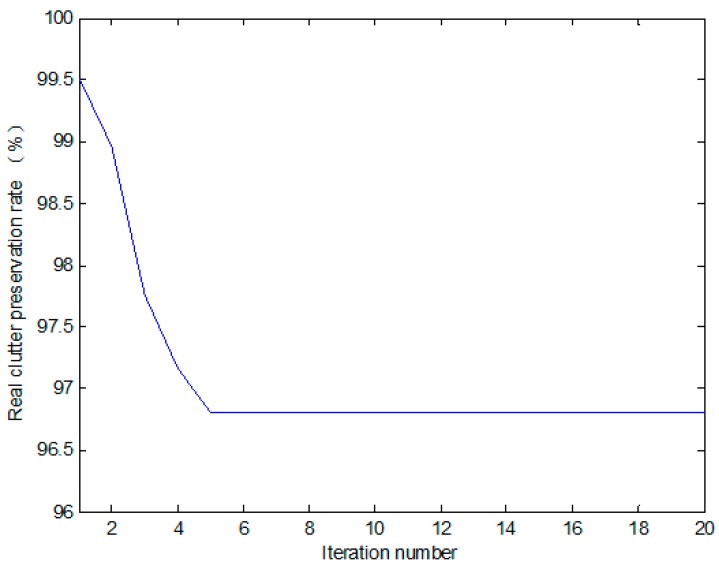
Relation between the real clutter preservation rate and the iteration number.

**Figure 13:**
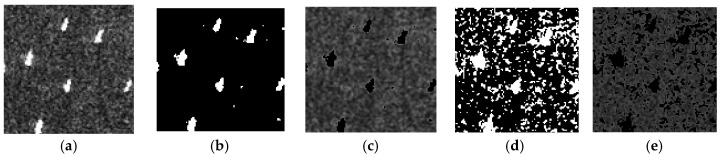
Clutter truncation result comparison: (**a**) the reference window image; (**b**) the removed pixels using an adaptive threshold of TS-2DLNCFAR with *t*_1_ = 1.9, and five iterations; (**c**) the truncated clutter by TS-2DLNCFAR; (**d**) the removed pixels by TS-CFAR with a truncation depth of 25%; and (**e**) the truncated clutter by TS-CFAR.

However, if $t_{1}$ is selected too high, one iteration cannot completely eliminate the high-intensity outliers in the background window, we can apply it iteratively. During each iteration, part of the high-intensity outliers can be eliminated, and the mean and standard deviation in the next iteration become smaller, so the high-intensity outliers preserved in the former iteration can be further eliminated. After several iterations, the high-intensity outliers can be eliminated completely, and the real clutter can be preserved to the largest degree. Figure 6 illustrates the relation between the real clutter preservation rate and the iteration number, where $t_{1}$ is set to 1.9 to preserve 97% of the real clutter. After five iterations, the outliers can be completely eliminated, and nearly 97% of the real clutter is preserved, which is shown in Figure 13b,c.

### 3.2. Parameter Estimation of the Truncated Clutter

Suppose the clutter samples in the local reference window are $X=\left\{X_{1},X_{2},\cdots X_{N_{c}}\right\}$, they all obey the same probability density function (PDF) $f_{X}\left(x\right)$ and cumulative distribution function (CDF) $F_{X}\left(x\right)$. After adaptive threshold-based clutter truncation, the truncated clutter samples become $\tilde{X}=\left\{{\tilde{x}}_{1},{\tilde{x}}_{2},\cdots {\tilde{x}}_{n}\right\}$. They obey the same PDF $f_{\tilde{X}}\left(x,t_{1}\right)$, and the PDF in the log-intensity domain of the truncated clutter $f_{\tilde{X}}\left(y=ln\left(x\right),t_{1}\right)$ can be expressed as:

(15) $$\begin{array}{l} f_{\tilde{X}}\left(y=ln(x),t_{1}\right)=\{\begin{array}{ll} \frac{f_{X}\left(x\right)}{F_{X}\left(\mu_{ln}+t_{1}\cdot \sigma_{ln}\right)}, & y\leq \mu_{ln}+t_{1}\cdot \sigma_{ln}\\ 0, & y>\mu_{ln}+t_{1}\cdot \sigma_{ln} \end{array}\\ =\{\begin{array}{ll} \frac{\exp\left(-{\frac{\left(ln(x)-\mu_{ln}\right)}{2\sigma_{ln}{}^{2}}}^{2}\right)}{\sqrt{2\pi }\sigma_{ln}x\cdot \left(\frac{1}{2}+\frac{1}{2}erf\left(\frac{t_{1}}{\sqrt{2}}\right)\right)}, & y\leq \mu_{ln}+t_{1}\cdot \sigma_{ln}\\ 0, & y>\mu_{ln}+t_{1}\cdot \sigma_{ln} \end{array} \end{array}$$

Then the mean and standard deviation in the log-intensity domain are estimated using the truncated clutter through the maximum likelihood estimator (MLE) [46]:

(16) $$\zeta \left(\mu_{ln},\sigma_{ln}|\tilde{X}\right)=\prod_{i=1}^{n}f_{\tilde{X}}\left({\tilde{x}}_{i}|\mu_{ln},\sigma_{ln}\right)=\frac{\exp\left(-\frac{\sum_{i=1}^{n}{\left(ln\left({\tilde{x}}_{i}\right)-\mu_{ln}\right)}^{2}}{2\sigma_{ln}{}^{2}}\right)}{{\left[\sqrt{2\pi }\sigma_{ln}\right]}^{n}\cdot \prod_{i=1}^{n}{\tilde{x}}_{i}\cdot {\left[\frac{1}{2}+\frac{1}{2}erf\left(\frac{t_{1}}{\sqrt{2}}\right)\right]}^{n}}$$

A logarithmic operation is applied to Equation (16), and the MLE is derived as:

(17) $$\frac{\partial ln\left[\zeta \left(\mu_{ln},\sigma_{ln}|\tilde{X}\right)\right]}{\partial \mu_{ln}}=0,\frac{\partial ln\left[\zeta \left(\mu_{ln},\sigma_{ln}|\tilde{X}\right)\right]}{\partial \sigma_{ln}}=0$$

The mean and standard deviation are estimated by:

(18) $${\hat{\mu }}_{ln}=\frac{1}{n}\sum_{i=1}^{n}ln\left({\tilde{x}}_{i}\right),\;{\hat{\sigma }}_{ln}=\sqrt{\frac{1}{n}{\sum_{i=1}^{n}\left(ln\left({\tilde{x}}_{i}\right)-{\hat{\mu }}_{ln}\right)}^{2}}$$

Similarly, the spatial correlation coefficients in the four directions using the adaptively-truncated clutter of the local reference window are defined as:

(19) $$r(l,k)=\frac{\sum_{\bar{i}=1}^{M}\sum_{\bar{j}=1}^{N}\left[f(\bar{i},\bar{j})-\bar{\mu }][f(\bar{i}+l,\bar{j}+k)-\bar{\mu }\right]}{\sum_{\bar{i}=1}^{M}\sum_{\bar{j}=1}^{N}{\left[f(\bar{i},\bar{j})-\bar{\mu }\right]}^{2}}$$

where $\left(\bar{i},\bar{j}\right)$, $\left(\bar{i}+l,\bar{j}+k\right)$ is included for spatial correlation coefficient estimation only under the condition that both of them belong to the truncated clutter. $\bar{\mu }$ is the gray intensity mean value of the truncated clutter of the local reference window.

### 3.3. Joint CFAR Detection and Fusion

The proposed TS-2DLNCFAR detector uses a 2DLN model as the JPDF of the truncated clutter. The detailed steps are specified as follows:

- Input the PFA, the sizes of the background window, and the test window.
- Obtain the truncated clutter using the adaptive threshold based clutter truncation introduced in Section 3.1.
- Estimate the mean ${\hat{\mu }}_{ln}$, standard deviation ${\hat{\sigma }}_{ln}$, and the spatial correlation coefficients in the four directions using the truncated clutter through Equations (18) and (19).
- Establish the JPDF in the four directions through Equation (1), and calculate the joint CFAR detection thresholds in the four directions through Equation (3).
- Joint CFAR detection is applied to the pixels under test in the test window using Equation (4).
- Let the local reference window slide on the SAR image, and obtain the four joint CFAR detection results in the four directions with a certain neighboring distance.
- The joint CFAR detection result of a certain neighboring distance is obtained by fusing the detection results in the four directions using the “OR” operation.
- All detection results of different neighboring distances are acquired, and the final detection result is obtained by fusing the detection results of different neighboring distances using the “AND” operation.

## 4. Experimental Results and Analysis

**Figure 7:**
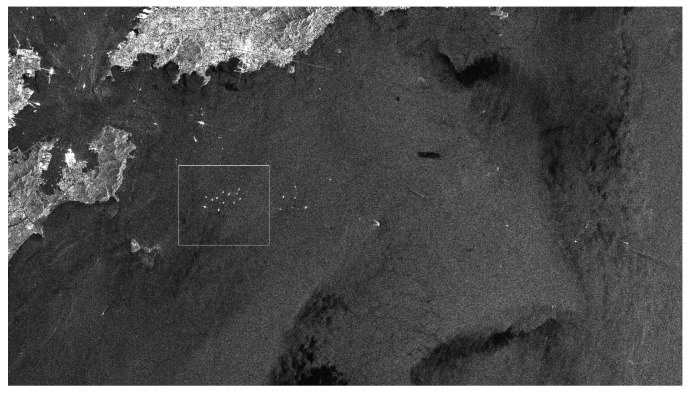
Low-resolution, multi-look, VV polarized SAR image of Qingdao harbor region acquired by the C-band Envisat-ASAR IM mode on 20 July 2007.

**Figure 8:**
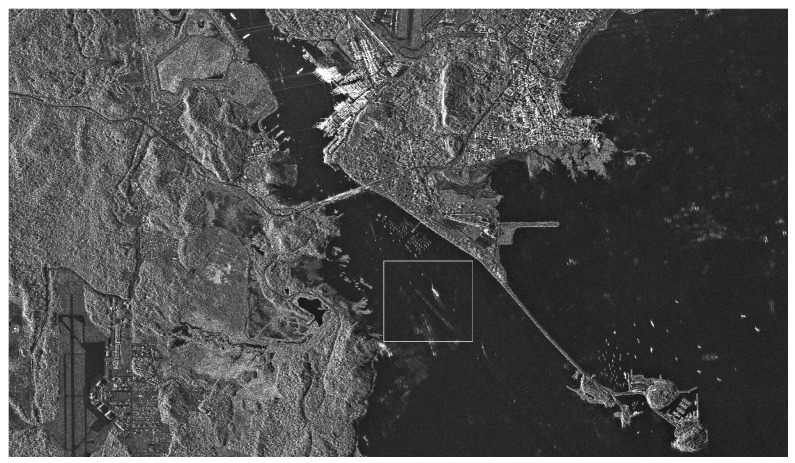
High-resolution, multi-look, HH polarized SAR image of the Panama Canal region acquired by the X-band TerraSAR SM mode on 31 July 2009.

In order to validate the excellent detection performance of the proposed TS-2DLNCFAR in high-intensity outliers’ presented areas, such as crowded harbors, the low-resolution, multi-look, VV polarized, C-band, amplitude Envisat-ASAR image (shown in Figure 7) and the high-resolution, multi-look, HH polarized, X-band, amplitude TerraSAR image (shown in Figure 8) are employed in this study. The Envisat-ASAR image was acquired by the IM mode on 20 July 2007 over the Qingdao harbor, of which the resolution is 30 m and the number of looks is 10. The TerraSAR-X image was acquired by the StripMap (SM) mode on 31 July 2009 over the Panama Canal, of which the resolution is 3 m and the number of looks is 50. We select the area of densely-distributed ship targets near the Qingdao harbor (the rectangle-marked area in Figure 7) and the ghosts and side-lobes presented area near the Panama canal (the rectangle marked area in Figure 8) to validate the detection performance. Since TS-CFAR outperforms other CFAR detectors in multiple target situations [42], TS-CFAR is selected to validate the better detection performance of the proposed TS-2DLNCFAR. Moreover, CA-CFAR [13], NM-CFAR [14], LN-CFAR [14], K-CFAR [10], and 2DLN-CFAR [15] are used for comparison to show the superiority of the proposed TS-2DLNCFAR. The experimental parameters are set as follows:

- For the low-resolution Envisat-ASAR image: CA-CFAR, NM-CFAR, LN-CFAR, and K-CFAR use a 41 × 41 reference window with a 21 × 21 guard window and a 1 × 1 test window. 2DLN-CFAR uses a 41 × 41 reference window with a 21 × 21 guard window and a 3 × 3 test window. TS-CFAR and the proposed TS-2DLNCFAR use a 41 × 41 reference window with no guard region, but TS-CFAR with a 1 × 1 test window and TS-2DLNCFAR with a 3 × 3, 5 × 5, 7 × 7, 9 × 9, and 11 × 11 test window.
- For the high-resolution TerraSAR-X image: CA-CFAR, NM-CFAR, LN-CFAR, and K-CFAR use an 81 × 81 reference window with a 41 × 41 guard window and a 1 × 1 test window. 2DLN-CFAR uses an 81 × 81 reference window with a 41 × 41 guard window and a 3 × 3 test window. TS-CFAR and the proposed TS-2DLNCFAR use an 81 × 81 reference window with no guard region, but TS-CFAR uses a 1 × 1 test window and TS-2DLNCFAR uses a 3 × 3, 5 × 5, 7 × 7, 9 × 9, and 21 × 21 test window.
- For TS-CFAR, the truncation depth is set to 25%, the same as that given by Ding et al. [42]. For the proposed TS-2DLNCFAR, the truncation degree $t_{1}$ is set to 1.9, and the iteration number is set to 5. Thus, the high-intensity outliers are eliminated, whereas 97% of real clutter samples are preserved for parameter estimation and statistical modeling.

**Figure 9:**
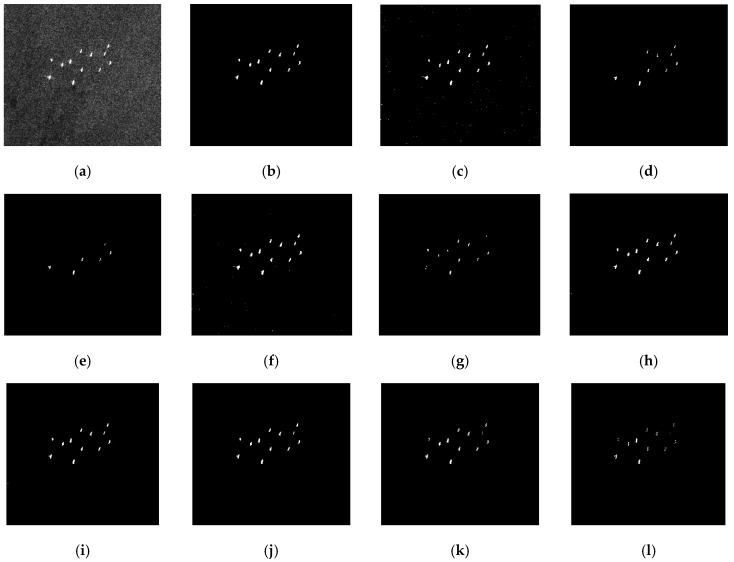
Detection performance comparison on a region of densely-distributed ship targets: (**a**) the multi-look Envisat-ASAR image, 12 targets are densely distributed; (**b**) ground truth; (**c**) NM-CFAR; (**d**) K-CFAR; (**e**) LN-CFAR; (**f**) TS-CFAR; (**g**) 2D-LNCFAR; and (**h**–**l**) are the detection results of the proposed TS-2DLNCFAR with a 3 × 3, 5 × 5, 7 × 7, 9 × 9, and 11 × 11 test windows, the truncation degree is set to 1.9. A 10^−4^ specified PFA is applied.

**Figure 10:**
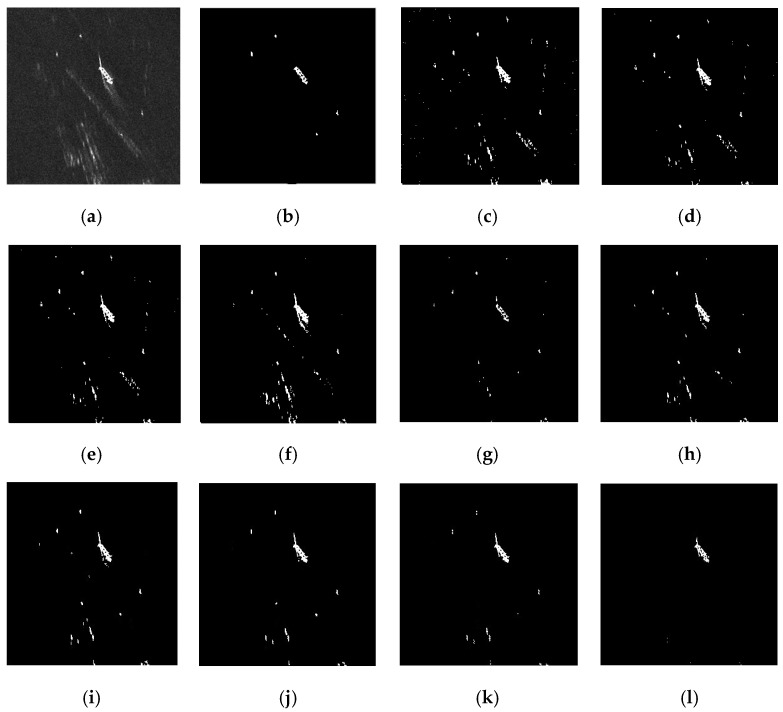
Detection performance comparison on ghosts and side-lobes in the presented region: (**a**) the multi-look TerraSAR-X image. One large ship, two small ship targets, and two “ghosts” are presented; (**b**) ground truth; (**c**) NM-CFAR; (**d**) K-CFAR; (**e**) LN-CFAR; (**f**) TS-CFAR; (**g**) 2D-LNCFAR; and (**h**–**l**) are the detection results of the proposed TS-2DLNCFAR with a 3 × 3, 5 × 5, 7 × 7, 9 × 9, and 21 × 21 test windows, the truncation degree is set to 1.9. A 10^−4^ specified PFA is applied.

A comparative analysis of the CFAR detectors is shown in Figure 9 and Figure 10 with a 10^−4^ specified PFA. The detection results of CA-CFAR are not shown because no pixel is detected. Figure 9a is the original Envisat-ASAR image (350 × 400), and there are 12 densely-distributed ship targets in the image. Traditional CFAR detectors such as CA-CFAR, K-CFAR, LN-CFAR, and 2DLN-CFAR use all pixels in the background window for parameter estimation, so the parameters are overestimated as a result of the interfering targets’ contamination. As a consequence, PD degrades dramatically, as shown. CA-CFAR misses all 12 targets, K-CFAR misses three, LN-CFAR misses six, 2DLN-CFAR misses one, and some detected ship targets are incomplete. NM-CFAR uses a Gaussian statistical model of the clutter, which cannot describe the long-tail property of the clutter. As a result, the FAR is unreasonably high, which reaches 0.252%, 14 dB larger than the given PFA. TS-CFAR and the proposed TS-2DLNCFAR can detect all of the ship targets using clutter truncation, but TS-CFAR has a FAR of 3 × 10^−4^ (42 false alarms) compared with 4 × 10^−5^ (five false alarms) of the proposed TS-2DLNCFAR with a 3 × 3 test window. With the increasing size of the test window, the FAR of the proposed TS-2DLNCFAR decreases. This is because the strong speckle is presented as isolated bright pixels, and ship targets are presented as a cluster of bright pixels with a certain length and width. Thus, when the neighboring distance becomes larger, TS-2DLNCFAR can exclude the false alarms effectively. However, if the selected size of the test window is too large, the PD degrades. In Figure 9h–j, all targets can be detected using 3 × 3, 5 × 5, and 7 × 7 test windows, however, when the size of the test window is larger than 7 × 7, some parts of the ship targets are missed, as shown in Figure 9k,l.

Figure 10a is the original TerraSAR-X image (280 × 280). There is one large ship, two small ships, and two “ghosts” caused by the low PRF. CA-CFAR again misses all the ship targets. NM-CFAR, K-CFAR, LN-CFAR, TS-CFAR, 2DLN-CFAR, and the proposed TS-2DLNCFAR can detect all of the ship targets. However, NM-CFAR suffers a high FAR, many clutter pixels are regarded as targets. LN-CFAR and K-CFAR suffer a relatively higher FAR, there are a large number of false alarms caused by side-lobes and ghosts. TS-CFAR suffers a higher FAR than LN-CFAR and K-CFAR for the exclusion of the high-intensity real clutter samples. 2DLN-CFAR and the proposed TS-2DLNCFAR comprehensively use the contrast and the correlation information, so they can detect all of the targets with a lower FAR. However, 2DLN-CFAR misses some parts of the large ship target. When the size of the test window is larger, the FAR of TS-2DLNCFAR becomes lower, while the PD is still high. However, when the size is as large as 21 × 21, all of the small targets are missed, whereas the large ship target can be detected. The proposed TS-2DLNCFAR achieves the best detection performance if the size of the test window is selected properly. In a real application, we can obtain the minimum size of the ships to be detected in the SAR image, and the best size of the test window can be selected as the minimum size of the ships to be detected. However, if the minimum size of the ship target is not known, comprehensively considering the PD and FAR, we can select a small size such as 5 × 5 or 7 × 7 to get a relatively promising detection result.

**Figure 11:**
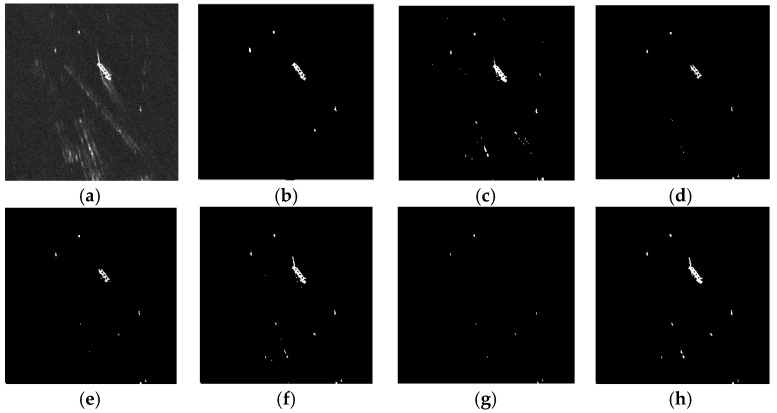
Detection performance comparison on ghosts and side-lobes in the presented region: (**a**) the multi-look TerraSAR-X image. One large ship, two small ship targets, and two “ghosts” are presented; (**b**) ground truth; (**c**) NM-CFAR; (**d**) K-CFAR; (**e**) LN-CFAR; (**f**) TS-CFAR; (**g**) 2D-LNCFAR; and (**h**) the proposed TS-2DLNCFAR with a 5 × 5 test window, and the truncation degree is set to 1.9. A 10^−9^ specified PFA is applied.

Since the PFA is not low enough, Figure 10 cannot demonstrate TS-2DLNCFAR’s superiority on high PD in ghosts and side-lobes’ presented backgrounds. Here, we lower the PFA to 10^−9^, and the detection results of the six CFAR detectors are shown in Figure 11. NM-CFAR still suffers a high FAR, and CA-CFAR misses all of the ships. K-CFAR misses one small ship target, LN-CFAR and K-CFAR miss some parts of the large ship target, and 2DLN-CFAR misses the large ship target. TS-CFAR and the proposed TS-2DLNCFAR with a 5 × 5 test window can detect all of the ship targets, but TS-2DLNCFAR has a lower FAR compared with TS-CFAR.

**Figure 12:**
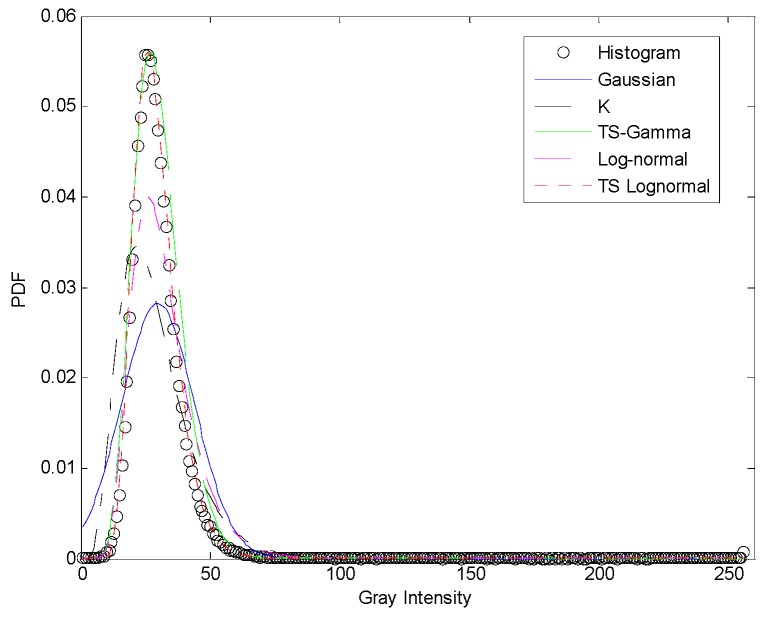
Goodness-of-fit test of different models. the black circles represent the histogram, the blue solid, the black dash-dotted, the green dashed, the pink dash-dotted, and the red dotted are Gaussian by NM-CFAR, K model by K-CFAR, truncated statistic-based Gamma by TS-CFAR, log-normal by LN-CFAR and 2DLN-CFAR, and adaptively-truncated statistics-based log-normal by the proposed TS-2DLNCFAR, respectively.

The detection performance is further verified by the goodness-of-fit test for each statistical model, as illustrated in Figure 12. Since the proposed TS-2DLNCFAR and 2DLN-CFAR use 2DLN distribution to model the JPDF of the sea clutter, it is not suitable to compare with the statistical models used by conventional CFAR detectors. However, we can use the log-normal model to show the better statistical modeling property through adaptive threshold-based clutter truncation. For CA-CFAR, LN-CFAR, K-CFAR, and 2DLN-CFAR, caused by the contamination of the interfering ship targets, the statistical models deviate from the histogram significantly. Both TS-CFAR and the proposed TS-2DLNCFAR apply clutter truncation firstly, so the statistical models are more accurate. However, the proposed TS-2DLNCFAR has a better fitting performance with a KL distance of 0.0289, compared with 0.1392 acquired by TS-CFAR. The above phenomenon can be described by the fact that 97% of the real clutter is preserved in the truncated clutter for the proposed TS-2DLNCFAR with a truncation degree of 1.9, while TS-CFAR discards a large amount of high-intensity real clutter with a truncation depth of 25%, which is shown in Figure 13. Although the estimation error of TS-CFAR is compensated by a normalization factor, the estimated parameters are still biased due to the incomplete clutter samples.

**Figure 14:**
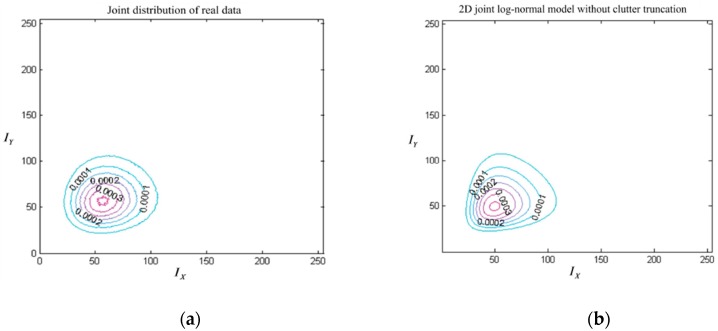

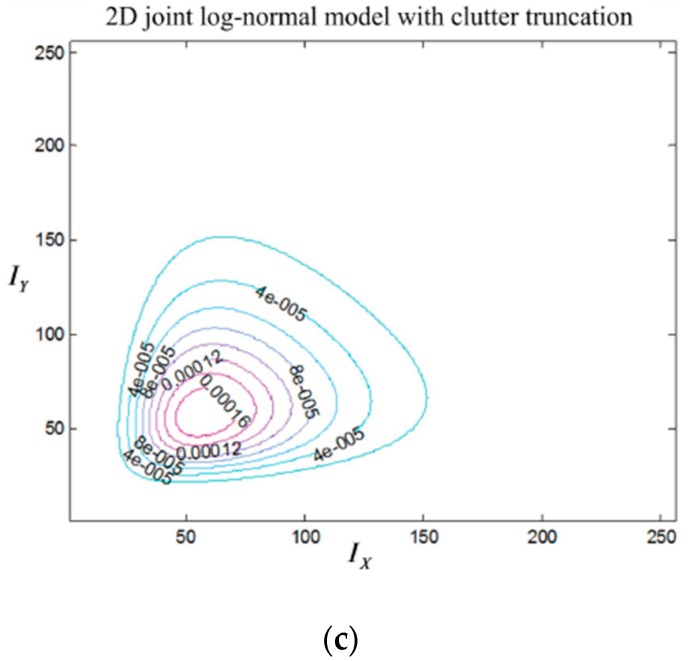
Goodness-of-fit test using the 2DLN model on real SAR data: (**a**) the joint distribution of the neighboring pixels in the horizontal direction; (**b**) 2DLN modeled from the truncated clutter by the proposed TS-2DLNCFAR; and (**c**) 2DLN modeled without clutter truncation by 2DLN-CFAR.

Furthermore, the 2DLN distribution modeled using all samples by 2DLN-CFAR is compared with that modeled by the proposed TS-2DLNCFAR using the adaptively-truncated clutter. Figure 14a shows the contour of the joint distribution using all of the clutter pixels in the background window, where the ship targets are excluded from the clutter samples. Figure 14b shows the contour of the 2DLN model based on the truncated clutter statistics by the proposed TS-2DLNCFAR, where the KL distance is 0.116. Figure 14c shows the 2DLN model using all of the pixels, including the interfering ship targets by 2DLN-CFAR, which deviates the actual JPDF of the clutter significantly, with a KL distance as large as 1.128. We can easily reach the conclusion that the 2DLN model based on the truncated clutter statistics has a better fitting performance compared with that without clutter truncation.

**Figure 15:**
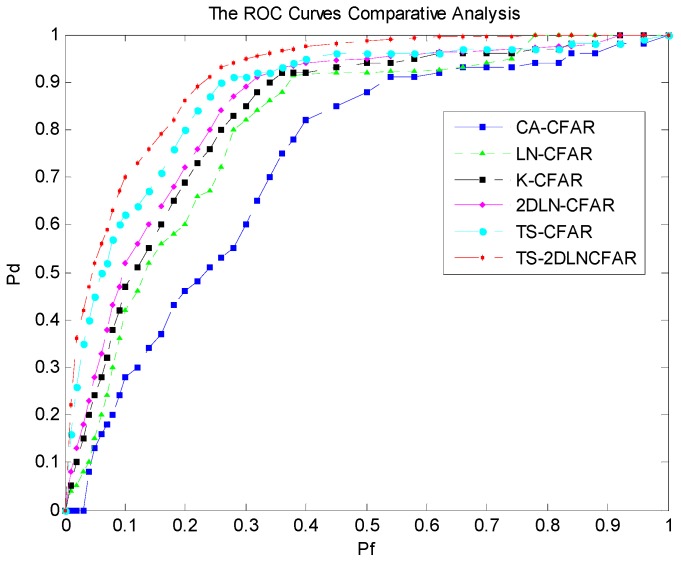
ROC curve comparative analysis: the blue dashed, the green dotted, the black dash-dotted, the pink dashed, the cyan dash-dotted, and the red dash-dotted are the ROC curves of CA-CFAR, LN-CFAR, K-CFAR, 2DLN-CFAR, TS-CFAR, and the proposed TS-2DLNCFAR with a 3 × 3 test window, respectively.

Furthermore, the receiver operating characteristic (ROC) curves are also derived to validate the detection performance of the proposed TS-2DLNCFAR with a 3 × 3 test window. The ROC curves are acquired using Monte Carlo simulations, where each simulation is conducted on the local reference window with a sample size of 1681 (41 × 41 window size including the PUT). The test image where a large number of ship targets are densely distributed is selected from Figure 7 with a size of 1020 × 1020. Firstly, the local reference windows are obtained with one pixel sliding step on the test image, and then the boundary pixels are discarded, so 1 × 10^6^ (1000 × 1000) local reference windows will be sent for Monte Carlo simulations. All six CFAR detectors are applied on each local reference window, and we can obtain the final detection binary map with different PFAs. The correctly-detected pixels and the falsely-detected pixels are counted from the detection binary image compared with the ground truth. Finally, the observed false alarm rate $P_{f}$, and the detection rate $P_{d}$ can be obtained through [47]:

(20) $$P_{f}=\frac{n_{fa}}{m\times n-n_{t}}$$

(21) $$P_{d}=\frac{n_{d}}{n_{t}}$$

where $n_{fa}$ are the observed false alarms, m and *n* are the length and width of the test image excluding the boundary pixels, both of which are 1000. *n_t_* is the true target pixel number, $m\times n-n_{t}$ is the real clutter pixel number of the test image. $n_{d}$ is the correctly detected pixel number. Figure 15 shows the derived ROC curves, where the target detection rate $P_{d}$ is plotted against the observed false alarm rate $P_{f}$. CA-CFAR, LN-CFAR, K-CFAR, and 2DLN-CFAR use all of the samples for statistical modeling, so the statistical models are quite inaccurate as a result of the high-intensity outliers’ contamination. As a consequence, the PD degrades. Both TS-CFAR and the proposed TS-2DLNCFAR apply clutter truncation firstly, so the statistical models established using the truncated clutter are more accurate than those without clutter truncation. TS-CFAR discards 25% of the high-intensity samples, and the statistical model is built using the 75% remaining low-intensity samples. However, the proposed TS-2DLNCFAR detector can preserve 97% of the real clutter while eliminating the high-intensity outliers, so the statistical model is more precise than that of TS-CFAR. TS-2DLNCFAR acquires the highest target detection rate under the same observed false alarm rate.

## 5. Conclusions

A correlation-based joint CFAR detector using adaptively-truncated statistics in log-normal clutter is proposed in this paper. The TS-2DLNCFAR detector exploits the gray intensity correlation information in ship targets and the clutter, and 2DLN distribution is built as the JPDF model of the sea clutter. The statistical model is accurately established through an adaptive threshold-based clutter truncation in the background window. Compared with traditional CFAR detectors, the statistical model is more accurate, and it acquires a higher PD in multiple target situations, such as crowded harbors and busy shipping lanes. Furthermore, the FAR is lower compared with other detectors, which has a great application value.

## References

[1] Leng X., Ji K., Zhou S., Xing X., Zou H. (2016). An adaptive ship detection scheme for spaceborne SAR imagery. Sensors.

[2] Wang W., Ji Y., Lin X., Xing X. (2014). A novel fusion-based ship detection method from pol-SAR images. Sensors.

[3] Li H., He Y., Wang W. (2009). Improving ship detection with polarimetric SAR based on convolution between co-polarization channels. Sensors.

[4] Gao G. (2011). A parzen-window-kernel-based CFAR algorithm for ship detection in SAR images. IEEE Geosci. Remote Sens. Lett..

[5] Amoon M. (2013). New method for ship detection in synthetic aperture radar imagery based on the human visual attention system. J. Appl. Remote Sens..

[6] Ai J., Qi X., Yu W., Deng Y., Liu F., Shi L. (2011). A novel ship wake CFAR detector based on SCR enhancement and normalized hough transform. IEEE Geosci. Remote Sens. Lett..

[7] Goldstein G.B. (1973). False-alarm regulation in log-normal and Weibull clutter. IEEE Trans. Aerosp. Electron. Syst..

[8] Oliver C.J. (1993). Optimum texture estimators for SAR clutter. J. Phys. D Appl. Phys..

[9] Li H.C., Hong W., Wu Y., Fan P. (2010). An efficient and flexible statistical model based on generalized Gamma distribution for amplitude SAR images. IEEE Trans. Geosci. Remote Sens..

[10] Oliver C.J. (1984). A model for non-Rayleigh scattering statistics. J. Mod. Opt..

[11] Wang C., Liao M., Li X. (2008). Ship detection in SAR image based on the alpha-stable distribution. Sensors.

[12] Frery A.C., Muller H., Yanasse C., Sant’Anna S. (1997). A model for extremely heterogeneous clutter. IEEE Trans. Geosci. Remote Sens..

[13] Finn H.M., Johnson R.S. (1968). Adaptive detection mode with threshold control as a function of spatially sampled clutter-level estimates. RCA Rev..

[14] Crisp D.J. (2004). The state-of-the-art in ship detection in synthetic aperture radar imagery. Org. Lett..

[15] Ai J., Qi X., Yu W., Deng Y., Liu F., Shi L. (2010). A new CFAR ship detector based on 2-D joint log-normal distribution in SAR images. IEEE Geosci. Remote Sens. Lett..

[16] Ritcey J. (1986). Performance analysis of the censored mean-level detector. IEEE Trans. Aerosp. Electron. Syst..

[17] Trunk G. (1978). Range resolution of targets using automatic detectors. IEEE Trans. Aerosp. Electron. Syst..

[18] Hansen V., Sawyers J. (1980). Detectability loss due to “Greatest of” selection in a cell-averaging CFAR. IEEE Trans. Aerosp. Electron. Syst..

[19] Smith M., Varshney P. (2000). Intelligent CFAR processor based on data variability. IEEE Trans. Aerosp. Electron. Syst..

[20] Gandhi P., Kassam S. (1988). Analysis of CFAR processors in homogeneous background. IEEE Trans. Aerosp. Electron. Syst..

[21] Huang X., Sun H., Luo W., Xu X., Yang W. (2004). Intelligent CFAR detector based on region classification for SAR images. J. Wuhan Univ. Nat. Sci. Ed..

[22] Himonas S., Barkat M. (1992). Automatic censored CFAR detection for non-homogeneous environments. IEEE Trans. Aerosp. Electron. Syst..

[23] Himonas S. (1992). Adaptive censored greatest-of CFAR detection. IEE Proc. Radar Signal Process..

[24] Conte E., Lops M., Tulino A. (1997). Hybrid procedure for CFAR in non-Gaussian clutter. IEEE Proc. Radar Sonar Navig..

[25] Bisceglie M., Galdi C. (2005). CFAR detection of extended objects in high-resolution SAR images. IEEE Trans. Geosci. Remote Sens..

[26] Gao G., Liu L., Zhao L., Shi G., Kuang G. (2009). An adaptive and fast CFAR algorithm based on automatic censoring for target detection in high-resolution SAR images. IEEE Geosci. Remote Sens. Lett..

[27] Shackelford A.K., Gerlach K., Blunt S.D. (2009). Partially adaptive STAP using the FRACTA algorithm. IEEE Trans. Aerosp. Electron. Syst..

[28] Magraner E., Bertaux N., Refregier P. (2010). Detection in gamma distributed non-homogeneous backgrounds. IEEE Trans. Aerosp. Electron. Syst..

[29] Cui Y., Zhou G., Yang J., Yamaguchi Y. (2011). On the iterative censoring for target detection in SAR images. IEEE Geosci. Remote Sens. Lett..

[30] An W., Xie C., Yuan X. (2014). An improved iterative censoring scheme for CFAR ship detection with SAR imagery. IEEE Trans. Geosci. Remote Sens..

[31] Farrouki A., Barkat M. (2005). Automatic censoring CFAR detector based on ordered data variability for non-homogeneous environments. IEEE Proc. Radar Sonar Navig..

[32] Song W., Wang Y., Liu H. (2016). An automatic block-to-block censoring target detector for high resolution SAR image. J. Electr. Inf. Technol..

[33] Zaimbashi A., Norouzi Y. (2008). Automatic dual censoring cell averaging CFAR detector in non-homogeneous environments. Signal Process..

[34] Chabbi S., Laroussi T., Barkat M. (2013). Performance analysis of dual automatic censoring and detection in heterogeneous Weibull clutter: A comparison through extensive simulations. Signal Process..

[35] Zaimbashi A. (2014). An adaptive cell averaging-based CFAR detector for interfering targets and clutter-edge situations. Digit. Signal Process..

[36] Tian S.R., Wang C., Zhang H. A segmentation based global iterative censoring scheme for ship detection in synthetic aperture radar image. Proceedings of the 2016 International Geoscience and Remote Sensing Symposium (IGARSS 2016).

[37] Ouchi K., Tamaki S., Yaguchi H., Iehara M. (2004). Ship detection based on coherence images derived from cross correlation of multilook SAR images. IEEE Geosci. Remote Sens. Lett..

[38] Brekke C., Anfinsen S.N., Larsen Y. (2013). Sub-band extraction strategies in ship detection with the sub-aperture cross-correlation magnitude. IEEE Geosci. Remote Sens. Lett..

[39] Dai H., Du L., Wang Y., Wang Z. (2016). A modified CFAR algorithm based on object proposals for ship target detection in SAR images. IEEE Geosci. Remote Sens. Lett..

[40] Yu W., Wang Y., Liu H., He J. (2016). Superpixel-based CFAR target detection for high-resolution SAR images. IEEE Geosci. Remote Sens. Lett..

[41] Rickard J., Dillard G. (1977). Adaptive detection algorithms for multiple target situations. IEEE Trans. Aerosp. Electron. Syst..

[42] Ding T., Anfinsen S., Brekke C. A Ship Detection Algorithm Based on Truncated Statistics. Proceedings of the 10th European Conference on Synthetic Aperture Radar Electronic Proceedings (EUSAR 2014).

[43] Ding T., Anfinsen S., Brekke C. (2016). Robust CFAR detector based on truncated statistics in multiple-target situations. IEEE Trans. Geosci. Remote Sens..

[44] Ding T., Anfinsen S., Brekke C. (2016). A Segmentation-Based CFAR Detection Algorithm Using Truncated Statistics. IEEE Trans. Geosci. Remote Sens..

[45] Brommundt J., B’ardossy A. (2007). Spatial correlation of radar and gauge precipitation data in high temporal resolution. Adv. Geosci..

[46] Robinson R. (1971). Gene Mapping in Laboratory.

[47] Fawcett T. (2006). An introduction to ROC analysis. Pattern Recognit. Lett..

